# Development of Sustainable Catalytic Pathways for Furan Derivatives

**DOI:** 10.3389/fchem.2021.707908

**Published:** 2021-11-22

**Authors:** Xiaofang Liu, Dayong Yu, Wenjia Yang, Qiuyun Zhang, Hongguo Wu, Can Li

**Affiliations:** ^1^ Guizhou Provincial Key Laboratory for Rare Animal and Economic Insects of the Mountainous Region, College of Biology and Environmental Engineering, Guiyang University, Guiyang, China; ^2^ School of Chemistry and Chemical Engineering, Anshun University, Anshun, China; ^3^ State-Local Joint Laboratory for Comprehensive Utilization of Biomass, Guizhou University, Guiyang, China

**Keywords:** polyoxometalates (POMs), zeolites, non-noble metal, ionic Liquids, furan derivatives

## Abstract

Biomass, the only globally available, renewable feedstock of organic carbon, is considered a viable alternative to fossil fuels. It can be efficiently utilized to produce various building blocks in accordance with green and sustainable chemistry principles. In this review, recent progress, such as the transformation of carbohydrates (C5 or C6 sugar, inulin, and cellulose) and their derivatives (furfural, hydroxymethylfurfural) into significant platform chemicals over polyoxometalates, zeolites, non-noble metals, and ionic liquids in single or multiphase, is evaluated.

## Introduction

Considerable effort must be devoted in transforming alternative and renewable resources to a form that would satisfy market requirements, industrial development, and remission environmental pressure given the increased consumption of nonrenewable resources and the environmental crises (such as global warming and chemical rain) (Tang et al., 2017). Biomass is the only globally available, abundant, and sustainable feedstock, and is considered a viable alternative to fossil fuels, which can be efficiently utilized for the preparation of various building blocks in accordance with green and sustainable chemistry. Soluble acids, which were previously used as catalysts for the conversion of biomass and its derivatives, are being discontinued because the complicated corrosion in the reaction contradicts green and sustainable principles. Nevertheless, fast mass transport, effective conversion of substrates, uniform texture, and good dispersion of the catalyst must be provided. This review summarizes the latest progress, such as the conversion of carbohydrates (C5 or C6 sugar, inulin, cellulose) and their derivatives [furfural, hydroxymethylfurfural (HMF) *via* hydrolysis, dehydration, hydrogenation, oxidation, hydrodeoxygenation, etc.] (Chheda et al., 2007; Fernandes et al., 2012; Gallezot, 2012) into significant platform chemicals (as shown in Table 1) over polyoxometalates, zeolites, non-noble metals, and ionic liquids in single or multiphase/s. The progress includes developments, such as catalysts affording tunable acidity and active sites, stable and uniform structure, and the excellent dispersion of catalytic centers to cater to sustainable requirements.

**TABLE 1:** Potential platform chemical applications.

Entry	Platform chemicals	Applications
1	Hydroxymethylfurfural (HMF)	Important precursor for polymers, fuels, or solvents
2	Levulinic acid (LA)	For the preparation of organic chemicals, polymers, resin, flavor agents, and fuel additives
3	5-ethoxymethylfurfural (EMF)	As a fuel additive, flavor, and aroma component additive
4	5-chloromethylfurfural (CMF)	Alternative chemicals, polymers, and intermediates for synthetic fuels
5	Alkyl levulinates (ALs)	Fuels and fuel additives, pharmaceuticals, food additives, agricultural products, solvents, and polymers
6	γ-valerolactone (GVL)	As green solvent, liquid fuel, food additive, precursor of renewable polymers, and for the manufacture of polymeric monomers, value-added chemical

## Sustainable Catalysts for Furan Derivatives

### Upgrading of Furan Derivatives Over Polyoxometalate-Based Catalysts

Polyoxometalates (POMs) are a special class of anionic polynuclear metal-oxo clusters with structural multiplicity and countercations balanced with a negative charge. Owing to their adjustable Brønsted or Lewis acidic nature and reducibility, POMs and POM-based materials have been widely used for heterogeneous catalysis (Wang and Yang, 2015; Enferadi-Kerenkan et al., 2018; Weinstock et al., 2018). For biomass valorization, POMs have been used as green acids or oxidation agents (Wang et al., 2015; Weinstock et al., 2018) to produce renewable chemicals and platform molecules, including industrial chemicals (Hu et al., 2015; Wang et al., 2015), organic acids, furans, (Bulushev and Ross, 2018; Wang et al., 2018; Zhang and Huber, 2018), and biodiesels (Narkhede et al., 2015). Similarly, metal–POM complexes have been applied to upgrade biomass into building block chemicals and fuels (Li et al., 2016). However, POMs are generally soluble in water and polar organic solvents; inorganic cations (e.g., K^+^, Sn^+^, Cs^+^, Ag^+^, etc.) usually serve as countercations to increase the insolubility of POMs. In addition, multiple heterogeneous uniform supports, including metal oxides, zeolites, porous silica, MOFs, and hybrids have been employed to immobilize POMs.

It is well known that HMF, furfuryl alcohol (FAL), and lactic acid are listed as significant platform chemicals in the “Top 10 + 4” list provided by the US Department of Energy (DOE) (Bozell and Petersen, 2010). A few compounds from the list will be discussed in this review. To date, various POM-based materials have been used as catalysts in HMF, LA, EMF, and ethyl levulinate (EL) production.

**FIGURE 1:**
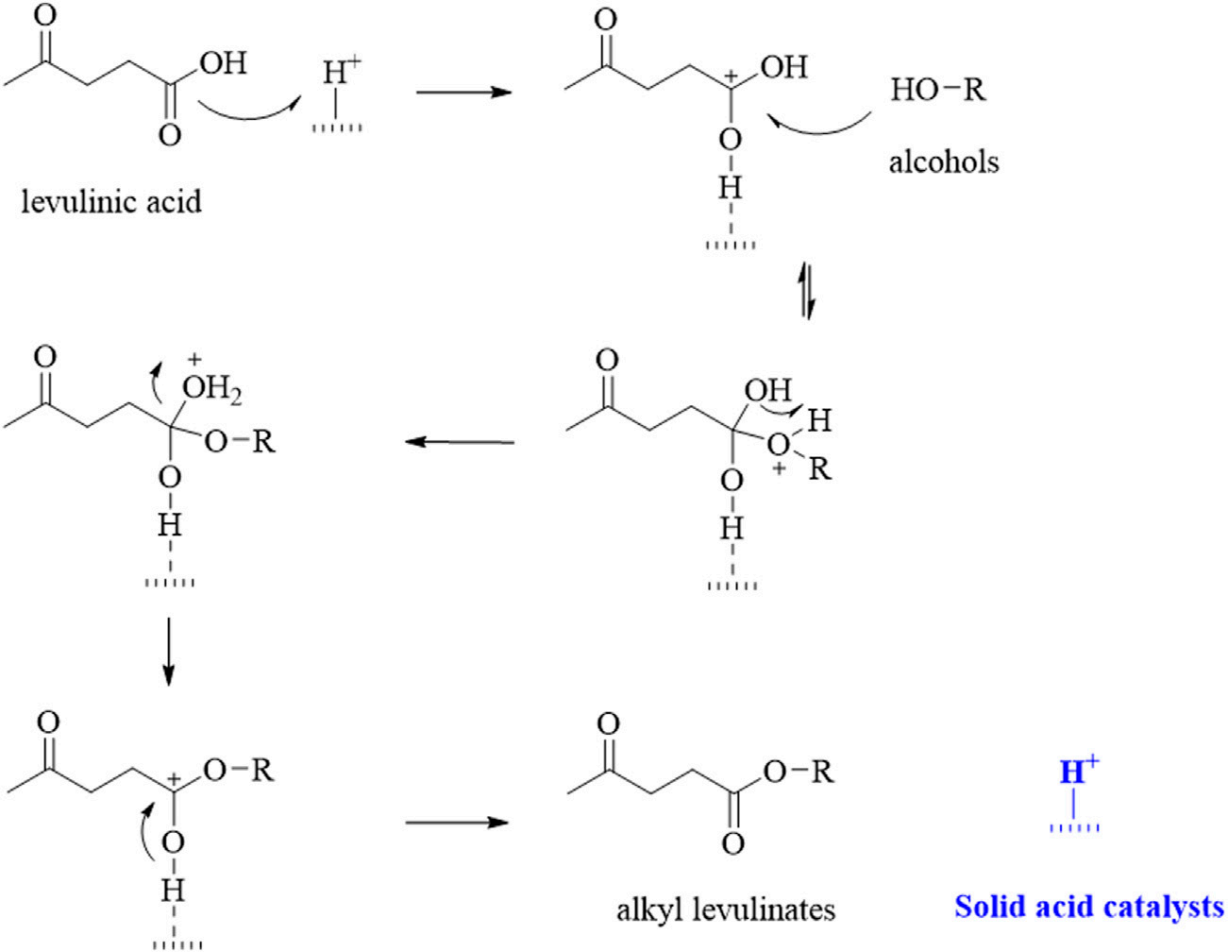
Plausible reaction mechanism of levulinic acid (LA) esterification with alcohol.

The LA esterification reaction for alkyl levulinate production has been examined over numerous heterogeneous solid acid catalysts that exhibit comparatively high efficiency (Pileidis et al., 2016; Cirujano et al., 2015). A plausible reaction mechanism for LA esterification with alcohols has also been proposed (Figure 1). A heteropolyacid (HPA) contains metal atoms such as W, Mo, and V, and *p*-block elements such as P, Si, etc.; the oxygen atoms connecting the metal atoms and H^+^ ions for charge balancing are generally used for recycling acidic catalysts for the synthesis of fine chemicals (Yan et al., 2013; Wu et al., 2016a; Wu et al., 2016b; Wu et al., 2016c; Zhou et al., 2016; Manikandan et al., 2017; Ramli et al., 2017; Zhang et al., 2017; Luan et al., 2018; Vilanculo et al., 2018; Lucas et al., 2019). Numerous structural analogs can be synthesized by either replacing the metal or nonmetal atoms. Furthermore, these HPAs possess ordered metal oxide nanoclusters, strong Brønsted (or Lewis) acidity and can be applied almost entirely as homogenous or heterogeneous catalysts, or reaction solvents (Zhou et al., 2016; Wu et al., 2016a; Ramli et al., 2017; Zheng et al., 2017).

Zhao et al. (2011) reported excellent catalytic activity and thermal stability for HMF production from pristine lignocellulosic biomass catalyzed by a Brønsted–Lewis-surfactant-combined HPA catalyst, Cr [(DS)H_2_PW_12_O_40_]_3_ (DS represents dodecyl sulfate, OSO_3_C1_2_H_25_). The result comprising 77.1% conversion of cellulose and 52.7% HMF yield is mainly ascribed to the synergistic acidities and micellar structure of the multihydrophobic groups. The stability and simple separation of the recycling catalyst demonstrate that micellar (HPA) Cr [(DS)H_2_PW_12_O_40_]_3_ satisfied green and sustainable principles. The aforementioned group synthesized a similar series of [HOCH_2_CH_2_N(CH_3_)_3_]_x_H_3−x_PW_12_O_40_ (Ch_x_H_3−x_PW_12_O_40_, x = 1, 2, and 3) with choline chloride and HPW, and achieved a high yield of up to 75% by ChH_2_PW_12_O_40_ at 140°C for 8 h (Zhang et al., 2016). The hydrophilic head of Ch_x_H_3−x_PW_12_O_40_ assembles the reactant cellulose, while the hydrophobic terminal suppresses the hydration side reaction of HMF into byproducts, thus, increasing the HMF yield.

Similarly, Sun et al. (2016) reported the original temperature-sensing HPA nanohybrid Ch_n_H_5−n_AlW_12_O_40_ catalysts that could be dissolved at high temperatures to afford a homogeneous reaction medium and removed from the catalytic system at room temperature. Among the explored catalysts, ChnH_5−n_AlW_12_O_40_ (*n* = 0–5) and ChH_4_AlW_12_O_40_ exhibited an excellent LA yield of 74.8% and 98.9% conversion of cellulose with green methyl isobutyl ketone (MIBK) as a co-solvent in a one-pot scheme, respectively; the results were attributed to a combination of temperature stimulus and Lewis–Brønsted double acidity. Additionally, these HPA catalysts can be easily reused without structural breakdown or loss of weight.

EMF is a potential alternative biofuel derived from biomass due to its high energy density (30.3 MJ·L^−1^), similar to that of regular gasoline (31.9 MJ·L^−1^) and comparable to that of diesel (33.6 MJ·L^−1^); EMF exhibits lower emissions of soot, NO_x_, and SO_x_ and, therefore, decreased contamination. Thus, EMF preparation from HMF or C5/C6 monose has drawn considerable attention (Liu and Wang, 2018). ALs has shown potential for use in fragrance and flavoring manufacturing, and as a mixture in biodiesel. EL is a novel diesel-blended biofuel.

**FIGURE 2:**
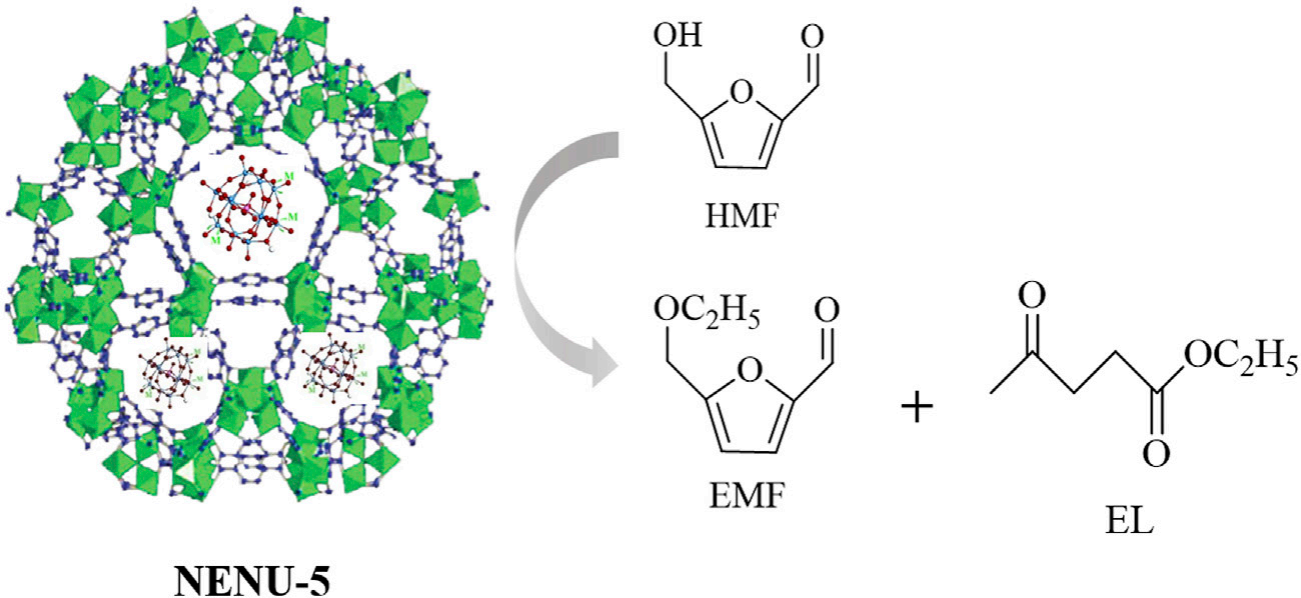
Schematic illustration of the catalysis procedure.

Wang et al. (2016) developed MOF-based POMs (Cu-BTC)(HPM) (NENU-5) [benzene-1,3,5-tricarboxylate (BTC) and phosphomolybdic acid hydrate (HPM)] to produce EMF and EL based on HMF (Figure 2). (Cu-BTC) (HPM) is composed of guest molecules of HPM that support the micropores of the stable and regular Cu-BTC host and exhibit notable catalytic performance and stability. The highest EMF and EL yields of 68.4% and 20.2% were observed at 140°C over 12 h, respectively.

Su et al. (2013) reported on ZrO_2_-based organic–inorganic hybrid materials functionalized by hydrophilic Keggin-type HPA and hydrophobic alkyl groups (i.e., benzene terminally bonded, and ethane-/benzene-bridged organosilica moieties), H_3_PW_12_O_40_/ZrO_2_-Si(Et/Ph)Si, and H_3_PW_12_O_40_/ZrO_2_-Si(Ph). The prepared catalysts exhibited good catalytic performance and stability for the esterification of LA with alcohols. The authors surmised that the excellent catalytic activity was due to the following factors: the strong Brønsted and Lewis collaborative acidity, unique textural properties, and hydrophobic groups that suppress the strong adsorption of the hydrophilic byproducts. In particular, the unique 2D hexagonal mesostructured H_3_PW_12_O_40_/ZrO_2_-Si(Ph)Si exhibited superior activity compared with the 3D wormhole-like H_3_PW_12_O_40_/ZrO_2_-Si(Et)Si and H_3_PW_12_O_40_/ZrO_2_-Si(Ph) because of the efficient diffusion in the regular mesoporous structure. The same authors examined HPA and ZrO_2_ bifunctionalized organosilica nanohybrid materials, e.g., PW_12_/ZrO_2_-Si(Et)Si with a 1D hollow tubular nanostructure, a 2D hexagonal periodic mesostructure, and a 3D interconnected mesostructure (Song et al., 2016). Among the materials examined, HPW and ZrO_2_-bifunctionalized organosilica nanotubes PW_12_/ZrO_2_-Si(Et)Si-NTs, exhibited the optimal performance for alkyl levulinates by LA esterification.

Furthermore, impressive metal/Si-pillared montmorillonite-supported heterophosphotungstate catalysts (SG-HPW-XSiMt-y, where X = La^3+^, Ce^3+^, Er^3+^, Cu^2+^, Al^3+^, Ti^4+^, Zr^4+^) were prepared for the synthesis of methyl levulinate (ML) from glucose (Lai et al., 2021). The study demonstrated that the appropriate Lewis acidity of the metal ions facilitated the key isomerization steps of glucose and methylglucoside. The strong Brønsted acidity of HPW suppressed the side reactions, and the mesoporous structure enhanced the catalytic activity, which allowed the surface and inside active sites to take effect. The optimum catalyst (SG-HPW-ZrSiMt-20) achieved the highest activity (65.8% yield of ML from glucose) at 170°C for 4 h. The SG-HPW-ZrSiMt-20 can be reused eight times without any obvious loss of catalytic activity owing to the uniform dispersion and stable bonding of HPW with the ZrSiMt support.

Various methods have been developed to transform POMs into reusable heterogeneous catalysts. However, leaching of these catalysts to the environment occurs, and further efforts are required to devise appropriate support materials. Furthermore, other unexplored POM structures (e.g., Lindqvist type) should be considered for application in biomass valorization.

### Zeolites as Catalysts for Upgrading Furan Derivatives

Zeolites with ordered micropores between 5 and 13 Å are usually highly structured crystalline aluminosilicates that exhibit superior thermal and chemical stability; most demonstrate Brønsted acidity. They assist the conversion of biomass substrates into platform molecules. Compared with microporous zeolites, mesoporous zeolites can afford excellent carrier materials with freedom from kinetic diffusion limitations in terms of larger reactants. Owing to their excellent structural features, high surface area, and relatively large pore size, mesoporous zeolites have been used to upgrade biomass for platform chemicals.

The design and preparation of zeolite catalysts that enhance product yield and afford good hydrothermal stability is a difficult and urgent problem that needs to be solved. This is evident from the reported literature on the conversion of biomass or biomass-derived platform chemicals.

It has been proven that zeolites are efficient for the dehydration of glucose to produce HMF. Mercedes explored a 57% conversion of glucose and a 1.6% yield of HMF in water at 195°C for 30 min; they employed H-ZSM-5 zeolites with a Lewis/Brønsted molar ratio of 0.25 as a catalyst. However, the application of a biphasic NaCl (20 wt%) aqueous solution/MIBK system by Moreno-Recio et al. (2016) achieved an enhanced conversion of 80% and an HMF yield of up to 42%. The group demonstrated that the catalytic activity was enhanced with the H-ZSM-5 zeolite by introducing NaCl in the biphasic reaction solvent, which is accredited with the inhibition of side reactions.

Based on the aforementioned research, Jiang et al. (2018) designed and prepared a zirconium-doped mesoporous KIT-6 catalyst (Zr-KIT-6) to transform glucose into HMF via dehydration. The highest glucose conversion (54.8%) and HMF yield (19.5%) were achieved at 170°C for 3 h in an aqueous phase. Similarly, the biphasic MIBK–water system increased the conversion of glucose to 79.0% and HMF yield to 34.5% under optimized conditions. This excellent catalytic activity is mainly ascribed to the high dispersion of ZrO_2_ nanoparticles and the existence of multicoordinated Zr^4+^ species in the Zr-KIT-6 materials.

Mesoporous SBA-15 materials are considered good candidates as supports for the dispersion of active sites because of their unique surface, pore texture with adjustable uniform hexagonal channels, and high hydrothermal stability (Verma et al., 2020). The group of Wu (Shi et al., 2011; Hua et al., 2013) constructed novel SBA-15-SO_3_H and AAO/SBA-15-SO_3_H catalysts processing active centers of sulfonic, carrier SBA-15, and substrates of porous alumina membranes (AAO). The xylose conversion and furfural selectivity were 90% and 70%, respectively, at 160°C for 4 h in a water/toluene two-phase reaction medium. The high reaction rate, mass transfer, and uniform distribution of the active sites in the developed AAO/SBA-15-SO_3_H catalyst contributed to its performance (Hua et al., 2013).

Ya’aini et al. (2013) assessed the LA yield of glucose valorization using a CrCl_3_/H-Y catalyst (1:1, w:w of CrCl_3_ and HY zeolite) composed mainly of hierarchical pores (i.e., mesopores) affected by the reaction temperature. The glucose conversion improved from 30% to 85% when the reaction temperature increased from 100°C to a maximum of 200°C for 3 h. Beyond the appropriate temperature of 140°C, decompositions of LA increased in the presence of HMF and formic acid (FA) (Antonyraj et al., 2014; Karwa et al., 2016; Liu et al., 2017; Piola et al., 2017; Wu et al., 2017); this explained the decrease in yield (Rackemann et al., 2014; Chen et al., 2018; Khan et al., 2018; Emel’yanenko et al., 2018). The results demonstrated that the type, quantity, and strength of the acid sites (Lewis acid), S_BET_, and hierarchical porous texture strongly influenced the catalytic activities during LA production. Further investigation revealed that the hybrid 1:1 catalyst afforded optimal catalytic activity with 62% LA yield at 160°C in 3 h.

Recently, the influence of the Sn/H-BEA catalyst on the glucose transformation of fructose and mannose was examined (Zhang et al., 2017). The conversion of the glucose substrate is generally enhanced by increasing the reaction temperature, and the maximum value of the conversion (up to 100%) may be attained because of the endothermic nature of the reaction (Saravanamurugan et al., 2013). The synthesis of glycosides was favored at higher reaction temperatures, whereas the production of fructose and methyl mannose declined markedly. The influence of temperature on the reaction performance can be attributed to the redistribution of the Sn species in the catalyst. A similar research was performed by Rajabbeigi et al. (2014), who studied the Sn/HBEA catalyst for the glucose upgrading isomerization of fructose at temperatures in the range 70–130°C and reached an analogous conclusion.

Further investigations could be performed involving the introduction of F^−^, P, and silane compounds to enhance the stability of zeolites (Jon et al., 2006; Louwen et al., 2020; Simancas et al., 2021). Additionally, research into extending the zeolite scope to contain natural materials with synthetic properties to accelerate the sustainability process should be considered.

### Upgrading of Furan Derivatives Catalyzed by Non-Noble Metals

The high price of noble metal catalysts limits their industrial application, and some non-noble metals that have similar chemical properties and catalytic activity, including Fe, Co, Ni, or Cu, have been appraised for biomass valorization. Non-noble metal catalysts can reduce the cost of catalysts; however, they have the problem of metal leaching.

Cerium exhibited fair catalytic activity in the hydrogenation reaction. Feng et al. (2019) developed a (CePO_4_)0.16/Co_2_P catalyst that achieved an LA conversion of 98.2% and GVL yield of 97.1% at 90°C, 4.0 MPa H_2_ for 1.5 h. Furthermore, the catalyst maintained excellent performance in five successive runs, which confirmed that it had long-term catalytic activity and stability under strongly acidic conditions.

Non-noble materials have been used more frequently for the hydrogenation of furfural. Gong et al. (2018) investigated two N-doped carbon nanotube-encapsulated metal nanoparticles, denoted as Ni@NCNTs-600–800 and Co@NCNTs-600–800, which displayed superior catalytic activity for furfural hydrogenation. The production of furfuryl alcohol was as high as 100% at 80°C, and active performance was maintained for six cycles. The excellent yield of further hydrogenation compounds containing tetrahydrofurfuryl alcohol or cyclopentanone suggested that the catalyst has excellent catalytic potential at higher temperatures and pressures.

In addition, non-noble metal oxides (Mn, Fe, Co, Ni, etc.) (Jiang et al., 2016; You et al., 2016, 2017; Schade et al., 2018) have been reported to synthesize carbon 1,4-diacids (FDCA). A high FDCA yield of up to 90% and robust stability were observed over NiCo_2_O_4_ nanowires (Gao et al., 2018). Non-noble metals (Fe–Zr–O) were introduced as a catalyst in (Bmim)Cl to realize the conversion of fructose to HMF. HMF was further oxidized to FDCA, and a yield of 46.4% was obtained with full fructose conversion under one-pot reaction conditions. Similarly, a potassium ferrate (K_2_FeO_4_) catalyst was developed, and a comparable optimal FDCA yield (48.3%) was obtained under the optimized reaction conditions (Zhang et al., 2015).

Yu et al. (2015) explored a carbon nanotube-loaded bimetallic Ni–Fe [(Ni–Fe/CNT, carbon nanotube (CNT)] catalyst for the preparation of *bis*(hydroxymethyl)furan (BHMF) and DMF derived from the selective hydrogenation and hydrogenolysis of HMF. The results indicated that Ni-Fe/CNT with a Ni/Fe atomic ratio of 2:1 exhibited high catalytic activity (TON 48) with enhanced production of BHMF (96%) at 110°C (91% DMF at 200°C), which is higher than that of monometallic Fe/CNTs. Unlike monometallic Fe/CNT, monometallic Ni/CNT exhibited high transformation (TON 34.7) at 110°C with low selectivity for target hydrogenated molecules, BHMF (76% at 110°C) and DMF (46% at 200°C). The results demonstrated that a combination of Ni and Fe in Ni–Fe/CNT displayed benefits for selective C–O bond cleavage.

In addition, Sheng and Lobo (2016) investigated the performance of Fe in the catalytic activity of Cu/SiO_2_ for furfural-2-MF (2-methylfuran) conversion, where they revealed that the Fe chemical state in the bimetallic Fe–Cu was significant in determining the selectivity; the uniform dispersion of the Cu particles was the primary reason for the excellent catalytic activity. Notably, the partial reduction of the Fe species in the pre-reduced Cu–Fe/SiO_2_ catalysts altered the selectivity of the target molecule from furfuryl alcohol to 2-MF based on furfural. Furthermore, the Cu species exhibited weak affinity toward the C=C bond when compared with the C=O bond; thus, Cu-based metallic catalysts showed high selectivity for the preparation of furfuryl alcohol (Sitthisa et al., 2011).

In addition, Guo et al., 2018 investigated the catalytic performance of NiCo–B active centers supported on acid-activated attapulgite for the synthesis of furfuryl alcohol via furfural hydrogenation (Guo et al., 2017; Guo et al., 2018). Owing to the strong synergistic effect between the Ni and Co active centers, the NiCo–B exhibited strong interactions with the acid-activated attapulgite (H^+^-ATP) carrier *via* the electronic charge effect between the bimetallic NiCo–B amorphous alloy and H^+^-ATP support. The excellent catalytic performance can be ascribed to the role of ATP in the dispersion of NiCo–B particles, adjustment of the partial charge distribution, and the strong synergistic effect between Co and Ni.

### Ionic Liquid-Catalyzed Conversion for Furan Derivatives

Ionic liquids (ILs) used in biomass valorization can be broadly divided into two categories, namely, 1) neutral ionic liquids and 2) functionalized ionic liquids. Neutral ionic liquids are normally used as solvents in biomass upgrading, whereas functionalized ionic liquids, generally including one or more acidic active groups, are used as solvents and catalysts. The meaningful conversion of C5 and C6 sugars to multipurpose green, renewable feedstocks, and fuel precursors illustrated the potential of ILs in biomass processing. Nevertheless, the efficient and convenient synthesis and recovery of ILs remain to be improved.

Acidic functionalized ILs catalyze the dehydration of carbohydrates to HMF and furfural in aqueous or alcohol media. The rehydration of HMF to afford LA or its derivatives is well known. Frequently used ILs, e.g., dimethylimidazolium hydrogensulfate (Kotadia and Soni, 2013; Eminov et al., 2014), 1-(4-butylsulfonic)-3-methylimidazolium hydrogensulfate (Matsagar et al., 2015; Wang et al., 2015), 1-(4-butylsulfonic)-3-allyl imidazolium hydrogensulfate, trifluoromethane sulfonate (Bao et al., 2008; Eminov et al., 2016), 1-carboxymethyl-3-methyl imidazolium chloride (Ma et al., 2015), and Cr^3+^ containing –SO_3_H functionalized polymeric BAILs (Li et al., 2013) have been extensively investigated as catalysts in the dehydration of monosaccharides to HMF under relatively mild conditions.

In addition to the dehydration of monosaccharides, ILs have been applied to the more complex multistep reactions of converting biomass to value-added platform molecules and feedstocks. For instance, a one-pot transformation of lignocellulosic and algal biomass into DMF was performed using a multicomponent catalytic system composed of *N*,*N*-dimethylacetamide methanesulfonate, Ru/C, and FA. (De et al., 2012).

LA or 4-oxopentanoic acid is a renewable generation platform molecule that can be obtained from C5 and C6 carbohydrates during dehydration and hydration reactions. This C5 keto-acid is generally used for the production of reproducible polymers and fuel precursors. The neutral and functionalized ILs acted as solvents or catalysts for the synthesis of LA and related derivatives (Tiong et al., 2018).

With 1-(3-propylsulfonic)-3-methylimidazolium chloride acting as the catalyst, a mixture of EL and LA was obtained from cellulose in aqueous ethanol via a one-pot successive process under mild conditions (Amarasekara and Wiredu, 2014). The optimal EL yield of 19.0% was achieved at 170°C, for 12 h, in a water–ethanol mixture medium containing 38.5% water. Furthermore, the highest LA yield (23.7%) was obtained for aqueous ethanol with a water content of 54% at 150°C for 48 h. The IL catalyst employed could be efficiently reused (96%) from the reaction medium (water) with negligible contamination.

ILs have also been employed as reaction media and catalysts for the conversion of biomass-derived derivatives. For instance, 90%–92% yield of succinic anhydride was obtained from HMF by oxidation with 30% H_2_O_2_ using 10 mol% 1-(alkylsulfonic)-3-methylimidazolium chloride acidic ILs as catalysts at 60°C for 14 h. The IL catalyst was recovered four times, with negligible loss in the catalytic performance. In addition, oxidation of furfural under similar reaction conditions afforded a mixture of three chemicals: 2(5H)-furanone, succinic anhydride, and maleic acid in 68%, 8%, and 12% yields, respectively (Amarasekara and Okorie, 2018).

## Upgrading of Furan Derivatives in Sustainable Reaction Media

**FIGURE 3:**
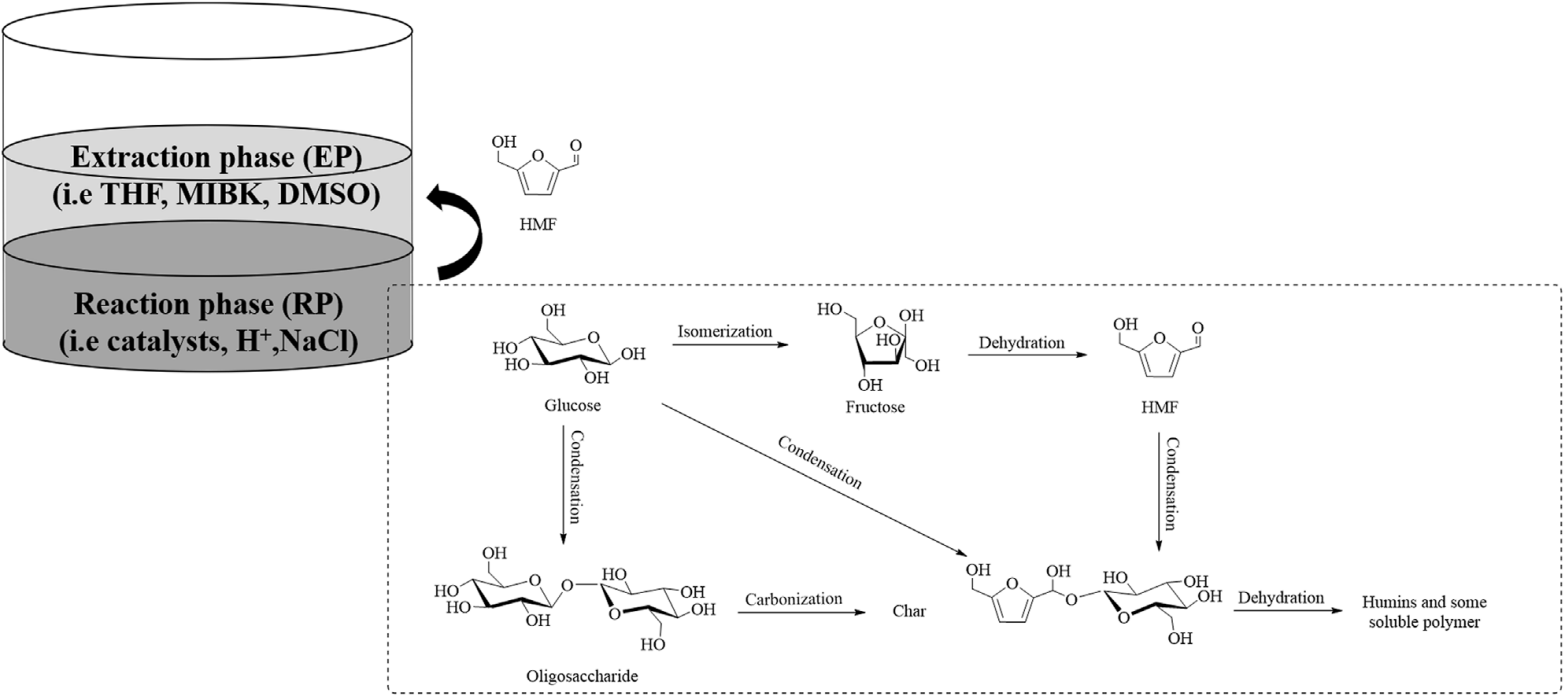
Undesired dehydration and condensation reactions from hydroxymethylfurfural (HMF).

Considering that the single reaction phase systems required the separation of the prepared derivatives, an energy intensive process and afforded low selectivity values for the target molecule, the development of an alternative multiphasic medium was necessary (Figure 3).

Tetrahydrofuran (THF) has been widely employed as the extraction phase (EP) solvent. At the appropriate reaction temperature of 140°C, glucose converted almost completely using phosphates as catalysts; CrPO_4_ exhibited a significantly higher selectivity of 63% to HMF (Xu et al., 2016) than that of FePO_4_ (23%) (Yang et al., 2015). Meanwhile, the reaction catalyzed by Cr-β zeolite at 150°C achieved a slightly lower conversion rate of 87%, but an increased selectivity of 83% (Xu et al., 2019). Published reports indicate that NaCl has been extensively utilized as a modifier for the reaction phase (RP). For example, hafnyl phosphates (HPs) have demonstrated high selectivity of up to 91% under the optimal reaction conditions of 175°C and 150 min, and the concentration of NaCl in the solvent was 4 wt% (Cao et al., 2019).

Methyl tetrahydrofuran (MeTHF) was selected to investigate the influence of a biphasic medium on the dehydration of glucose at 175°C for 80 min using phosphate TiO_2_ as a catalyst. Comparing the biphasic system effect with the catalytic performance using MeTHF-N-methyl pyrrolidone (NMP) (v:v, 6:1), a notable enhancement of glucose conversion from 89.4% to 97.9% and HMF yield from 58.8% to 90.7% was observed (Atanda et al., 2016). The Atanda group explored using dioxane as EP, comparing the catalytic performance of other solvents containing THF, 1-BuOH, MIBK, and 1-propanol, all of which had a consistent NaCl content (20 wt%). With the hybrid catalysts composed of TiO_2_–ZrO_2_ and Amberlyst 70, almost full conversion of glucose and 86% product yield was achieved at 175°C for 3 h (Atanda et al., 2015).

In the interim, a combination of H_2_O-DMSO (3:1, w/w)/MIBK–1-BuOH (7:3, w/w) was employed in a reaction medium, in which a modified Cr(III)-containing polydivinylbenzene polymeric material with bifunctional Brønsted–Lewis acidity was utilized as the catalyst, affording 95% glucose conversion and 59% HMF yield (Wang et al., 2016). In addition, the same glucose–HMF reaction system was researched using zeolite β as a catalyst in the H_2_O–DMSO (9:1 v/v)/THF biphasic system at 180°C for 30 min, and a relatively high conversion of 80% and selectivity of 75% were obtained (Otomo et al., 2015).

**FIGURE 4:**
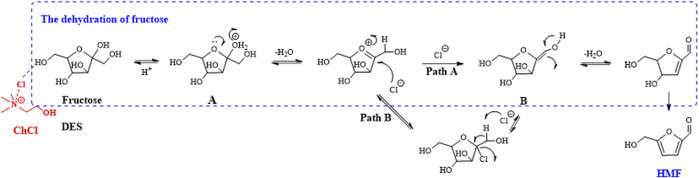
Possible synthesis mechanism of HMF from fructose in the deep eutectic solvents (DES)/acetonitrile biphasic system.

In contrast to biphasic media, some researchers have concentrated on the use of deep eutectic solvents (DESs) as reaction media. An interesting composition was constructed using tetraalkyl ammonium chloride and alkylamine hydrochloride salts for fructose transformation (Figure 4). The salts were used as the catalyst, RP with NaHSO_4_ acted as the co-catalyst, and THF was utilized as the EP; these reaction conditions provided the maximum HMF yield of 83% at 120°C (Cao et al., 2011). Recently, a similar DES system was reported; it was composed of choline chloride (ChCl) as a hydrogen bond acceptor (HBA) and the reactant fructose as a hydrogen bond donor (HBD) (molar ratio 5:1), with acetonitrile as the EP. The results showed that complete conversion of fructose was achieved with 90% HMF yield using HCl as a catalyst after 4 h at 100°C (Zuo et al., 2017).

A biphasic reaction medium was also studied for polysaccharide conversion to HMF. For example, the mixed reaction media H_2_O/2-BuOH was utilized *in situ* for the inulin transformation with a catalyst dosage of 0.5 wt% of pretreated niobium pentoxide in the RP and gave a 54% yield in 140 min (Yang et al., 2011). The catalytic conversion reaction was also tested in the ChCl:oxalic acid DES reaction medium, simultaneously, with EtOAc as EP (v:v, 1:10), obtaining 64% conversion of inulin with complete selectivity for the synthesis of HMF (Hu et al., 2009). Based on the previously described work, the different species of RP and EP for the same reaction with inulin as a reactant, the optimal result with H_2_O–NMP (4:6, w/w)/MIBK, offered a 69% yield. In addition, sucrose was explored to produce an HMF-verifying solvent system, affording a maximum yield of 43%, although the conversion of sucrose was relatively low at only 58% (Chheda and Dumesic, 2007).

A more interesting alternative for the preparation of furans in EP–RP systems could be the production of CMF. The systematic research of the synthesis of CMF from microcrystalline cellulose, corn stover, straw, or birch wood was performed with an H_2_O/dichloroethane solvent pair modified with LiCl (Mascal and Nikitin, 2008; Mascal and Nikitin, 2009).

In addition, *in situ* extraction for the target chemical furfural was attempted with supercritical CO_2_, for which the conditions of 20 MPa, 150°C, and CO_2_ flow rate of 3.77 g min^−1^ were needed to achieve 88% conversion of xylose degradation and 52% yield with a 10-wt% dosage of Amberlyst 70 as a catalyst (Sato et al., 2019).

## Summary and Outlook

Recent advances in the chem-catalytic valorization of biomass and derivatives into chemicals and fuels using green and sustainable approaches have been summarized. The heterogeneous solid catalysts, POM, zeolites, non-noble metals, and ILs are environmentally friendly materials. Additionally, the multiphasic extract affords efficient sustainable utilization of the reaction medium and catalyst and reduces resource requirements, which are needed for the separation of products.

Despite the aforementioned developments, there are still considerable challenges and opportunities for biomass upgrading using inexpensive, green, and efficient heterogeneous catalysts. First, leaching still exists, thus, compromising catalyst stability. Future efforts should be devoted to the investigation of substrate–catalyst interactions and the kinetics and thermodynamics of various catalytic materials. Second, a deeper understanding of the relationship between the catalytic performance and the chemical/electronic/structural properties of catalytic materials will be important for biomass valorization. Furthermore, the choice of the catalytic system is the most challenging problem, and determines catalyst reusability and the potential for industrial applications.

## Abbreviations

DES, deep eutectic solvents; DMF, 2,5-dimethylfuran; EL, ethyl levulinate; EMF, 5-ethoxymethylfurfural; EP, extraction phase; FA, formic acid; FDCA, furan dicarboxylic acid; HBA, hydrogen bond acceptor; HBD, hydrogen bond donor; HMF, hydroxymethylfurfural; HP, hafnyl phosphates; HPA, heteropolyacid; HPM, phosphomolybdic acid hydrate; IL, ionic liquid; LA, levulinic acid; MeTHF, methyl tetrahydrofuran; MIBK, methyl isobutyl ketone; ML, methyl levulinate; POM, polyoxometalate; RP, reaction phase.
